# Supplementary material for: Deletion of wfs1 Impairs Oligodendrocyte Precursor Cells Dorsal Distribution and Myelination Through the wfs1-hmgcs1 Axis in Zebrafish
Source: Biology (Basel). 2026 Aug 16;15(16):1402. doi: 10.3390/biology15161402 (PMC13510151; doi:10.3390/biology15161402)
Supplement: Supplementary file 1 [file biology-15-01402-s001.zip › File S1-Transcriptome sequencing data and differentially expressed genes (DEGs) in wfs1-- zebrafish at 5 dpf/2.KEGG/wfs1KOvsWT/GSEA/wfs1KO/FANCONI_ANEMIA_PATHWAY/FANCONI_ANEMIA_PATHWAY(DRE03460).html]

Details for gene set FANCONI\_ANEMIA\_PATHWAY(DRE03460)[GSEA]

|  || Dataset | deg.sample |
| Phenotype | sample.cls |
| Upregulated in class | wfs1KO |
| GeneSet | FANCONI\_ANEMIA\_PATHWAY(DRE03460) |
| Enrichment Score (ES) | 0.35949376 |
| Normalized Enrichment Score (NES) | 1.3184804 |
| Nominal p-value | 0.028 |
| FDR q-value | 0.27131552 |
| FWER p-Value | 0.946 |
Table: GSEA Results Summary

  

Fig 1: Enrichment plot: FANCONI\_ANEMIA\_PATHWAY(DRE03460)      
 Profile of the Running ES Score & Positions of GeneSet Members on the Rank Ordered List

  

| PROBE | DESCRIPTION (from dataset) | GENE SYMBOL | GENE\_TITLE | RANK IN GENE LIST | RANK METRIC SCORE | RUNNING ES | CORE ENRICHMENT || 1 | 558923 | palb2 |  |  | 241 | 3.937 | 0.0774 | Yes |
| 2 | 100538153 | slx4 |  |  | 384 | 3.331 | 0.1447 | Yes |
| 3 | 402936\_1 | fancg |  |  | 755 | 2.462 | 0.1862 | Yes |
| 4 | 406487 | rad51 |  |  | 855 | 2.308 | 0.2329 | Yes |
| 5 | 794038 | brip1 |  |  | 873 | 2.280 | 0.2815 | Yes |
| 6 | 570043\_1 | rev1 |  |  | 1086 | 2.009 | 0.3182 | Yes |
| 7 | 450081 | rad51c |  |  | 1739 | 1.512 | 0.3303 | Yes |
| 8 | 565458\_1 | fan1 |  |  | 2245 | 1.268 | 0.3418 | Yes |
| 9 | 100006685 | fancm |  |  | 2491 | 1.177 | 0.3595 | Yes |
| 10 | 777754\_1 | wdr48a |  |  | 3270 | 0.979 | 0.3562 | No |
| 11 | 565458 | fan1 |  |  | 6717 | 0.518 | 0.2591 | No |
| 12 | 565276 | pms2 |  |  | 7515 | 0.453 | 0.2438 | No |
| 13 | 566857\_1 | poln |  |  | 7729 | 0.440 | 0.2466 | No |
| 14 | 393149 | rmi1 |  |  | 8303 | 0.412 | 0.2375 | No |
| 15 | 566758 | brca2 |  |  | 8769 | 0.383 | 0.2312 | No |
| 16 | 394241 | fancd2 |  |  | 8870 | 0.376 | 0.2361 | No |
| 17 | 678520\_1 | polh |  |  | 9177 | 0.354 | 0.2342 | No |
| 18 | 322007 | usp1 |  |  | 9207 | 0.353 | 0.2409 | No |
| 19 | 768152 | ube2t |  |  | 9593 | 0.327 | 0.2358 | No |
| 20 | 100001845 | slx1b |  |  | 9765 | 0.318 | 0.2373 | No |
| 21 | 558534 | atrip |  |  | 9984 | 0.304 | 0.2370 | No |
| 22 | 393632 | mlh1 |  |  | 10059 | 0.300 | 0.2411 | No |
| 23 | 793414 | top3b |  |  | 10620 | 0.265 | 0.2293 | No |
| 24 | 65226 | rpa2 |  |  | 10674 | 0.262 | 0.2333 | No |
| 25 | 140613\_1 | her9 |  |  | 10832 | 0.253 | 0.2338 | No |
| 26 | 334682 | wdr48b |  |  | 11831 | 0.199 | 0.2067 | No |
| 27 | 565276\_1 | pms2 |  |  | 12414 | 0.168 | 0.1921 | No |
| 28 | 324185 | fanci |  |  | 12572 | 0.161 | 0.1906 | No |
| 29 | 327322 | ercc4 |  |  | 13473 | 0.117 | 0.1648 | No |
| 30 | 565685 | fance |  |  | 13518 | 0.115 | 0.1659 | No |
| 31 | 560212 | top3a |  |  | 13519 | 0.115 | 0.1684 | No |
| 32 | 692127\_1 | fancc |  |  | 13570 | 0.112 | 0.1693 | No |
| 33 | 777634 | telo2 |  |  | 15269 | 0.034 | 0.1166 | No |
| 34 | 569270\_1 | apitd1 |  |  | 16092 | -0.005 | 0.0909 | No |
| 35 | 692127 | fancc |  |  | 16301 | -0.011 | 0.0846 | No |
| 36 | 393196 | rpa3 |  |  | 16744 | -0.024 | 0.0712 | No |
| 37 | 572540 | blm |  |  | 17101 | -0.036 | 0.0608 | No |
| 38 | 558350 | eme1 |  |  | 17156 | -0.039 | 0.0600 | No |
| 39 | 327491 | rpa1 |  |  | 17594 | -0.058 | 0.0475 | No |
| 40 | 100332714 | stra13 |  |  | 17915 | -0.072 | 0.0390 | No |
| 41 | 567770 | atr |  |  | 18166 | -0.084 | 0.0330 | No |
| 42 | 100001169 | si:dkey-57h18.1 |  |  | 18301 | -0.089 | 0.0307 | No |
| 43 | 559470 | faap24 |  |  | 18966 | -0.120 | 0.0124 | No |
| 44 | 406839 | mus81 |  |  | 20943 | -0.212 | -0.0451 | No |
| 45 | 550532 | poli |  |  | 21235 | -0.227 | -0.0494 | No |
| 46 | 777754 | wdr48a |  |  | 21450 | -0.238 | -0.0509 | No |
| 47 | 692250 | fancf |  |  | 22352 | -0.287 | -0.0731 | No |
| 48 | 30288\_1 | her6 |  |  | 23038 | -0.330 | -0.0875 | No |
| 49 | 692125 | fanca |  |  | 23845 | -0.383 | -0.1045 | No |
| 50 | 100001425 | ercc1 |  |  | 24484 | -0.419 | -0.1155 | No |
| 51 | 406255 | fancl |  |  | 24491 | -0.419 | -0.1067 | No |
| 52 | 563218 | rmi2 |  |  | 24602 | -0.422 | -0.1011 | No |
| 53 | 793414\_1 | top3b |  |  | 25039 | -0.449 | -0.1051 | No |
| 54 | 569100 | rev3l |  |  | 26297 | -0.557 | -0.1325 | No |
| 55 | 406487\_1 | rad51 |  |  | 26690 | -0.598 | -0.1320 | No |
| 56 | 100332307 | eme2 |  |  | 26909 | -0.621 | -0.1254 | No |
| 57 | 562749 | polk |  |  | 27696 | -0.700 | -0.1350 | No |
| 58 | 566857 | poln |  |  | 28525 | -0.826 | -0.1432 | No |
| 59 | 570043 | rev1 |  |  | 28874 | -0.894 | -0.1349 | No |
| 60 | 100538153\_1 | slx4 |  |  | 31307 | -2.447 | -0.1585 | No |
| 61 | 558923\_1 | palb2 |  |  | 31402 | -2.651 | -0.1043 | No |
| 62 | 794038\_1 | brip1 |  |  | 31448 | -2.767 | -0.0460 | No |
| 63 | 402936 | fancg |  |  | 31450 | -2.776 | 0.0138 | No |
Table: GSEA details [plain text format]

  

Fig 2: FANCONI\_ANEMIA\_PATHWAY(DRE03460)      
 Blue-Pink O' Gram in the Space of the Analyzed GeneSet

  

Fig 3: FANCONI\_ANEMIA\_PATHWAY(DRE03460): Random ES distribution      
 Gene set null distribution of ES for **FANCONI\_ANEMIA\_PATHWAY(DRE03460)**
